# Clinical Characteristics and Treatment of Juvenile Myasthenia Gravis—A Single-Center Experience

**DOI:** 10.3390/children11050572

**Published:** 2024-05-09

**Authors:** Mikiko Maeda, Hideki Shimomura, Sachi Tokunaga, Naoko Taniguchi, Tomoko Lee, Yasuhiro Takeshima

**Affiliations:** Department of Pediatrics, Hyogo Medical University School of Medicine, 1-1, Mukogawa, Nishinomiya 663-8501, Japan; mikeah.105@icloud.com (M.M.); sa-tokunaga@hyo-med.ac.jp (S.T.); nao-taniguchi@hyo-med.ac.jp (N.T.); to-ri@hyo-med.ac.jp (T.L.); ytake@hyo-med.ac.jp (Y.T.)

**Keywords:** drug treatment, immunosuppressants, juvenile myasthenia gravis, outcome, prednisolone

## Abstract

Juvenile myasthenia gravis (MG) is a rare autoimmune neuromuscular disease, often treated with anticholinesterases, corticosteroids, and immunosuppressants. However, optimal treatment durations remain unclear. This study investigated the clinical characteristics and treatment of juvenile MG, including medication duration. The administration period for all drugs, immunosuppressants, and prednisolone at doses greater than 0.35 mg/kg daily was extracted retrospectively from medical records. Nineteen participants (8 boys, 11 girls) aged 8 months to 14 years (median, 2.5 years) at onset were identified. Fourteen patients (73.7%) had ocular MG and five (26.3%) had generalized MG. Drug treatment was conducted in 18 cases; however, 7 patients did not complete the treatment. Among the patients who completed drug treatment, the duration of treatment ranged from 11 to 100 months (median, 47 months). In the six patients treated with continuous administration of prednisolone or immunosuppressants, the treatment duration ranged from 33 to 99 months (median, 56 months). No severe adverse effects requiring hospitalization were reported. The patients treated with prednisolone or immunosuppressants required at least 33 months of treatment. These results will help develop protocols for juvenile MG treatment.

## 1. Introduction

Myasthenia gravis (MG) is an autoimmune neuromuscular disorder with neuromuscular transmission dysfunction, characterized by weakness or fatigue involving the ocular, bulbar, limb, or respiratory muscles [1]. Depending on whether only ocular or systemic symptoms develop, it is divided into the ocular-type MG (OMG) and generalized-type MG (GMG) [1]. Although MG can develop at any age, onset peaks in the third and sixth decades, predominantly in females [1]. Juvenile MG represents childhood onset and has characteristics different from adults [2]. Juvenile MG is a rare disease that accounts for approximately 10–15% of all MG cases. It has characteristics such as more antibody-negative cases, exists more commonly as the ocular type compared to that in adults [3,4,5,6], and has spontaneous remission [7].

Anticholinesterases, corticosteroids, and immunosuppressants are used for treatment, and the treatment period is often prolonged when corticosteroids or immunosuppressants are used [8]. Moreover, most patients experience fluctuating symptoms or recurrence [7,9], requiring long-term treatment. In particular, when corticosteroids are used in children, there are concerns regarding the adverse effects of long-term administration, including cataracts, glaucoma, steroid myopathy, psychosis, and growth suppression. Especially in children, short stature caused by growth suppression has been a major concern [10].

This may be owing to rare diseases and long treatment periods, and there have been few detailed reports regarding the course of treatment and duration of medication [6,11]. Several reports have suggested that when corticosteroids are used for a long duration, switching to immunosuppressants should be considered to reduce the side effects [8,12,13]. However, the appropriate administration period for corticosteroids and immunosuppressants has not yet been established. This study aimed to summarize the clinical characteristics of juvenile MG and assess the outcomes, including the duration of medication.

## 2. Materials and Methods

### 2.1. Participants

We retrospectively analyzed the medical records of all patients diagnosed with juvenile MG between 2005 and 2022 at the Pediatric Department of Hyogo Medical University Hospital, Japan. 

The diagnosis of OMG was based on the manifestations of fatigable muscle weakness, including ptosis, diplopia, or both, which were improved by the edrophonium test. The diagnosis of GMG was based on manifestations of muscle weakness involving a variable combination of bulbar, limb, and respiratory muscles and positive findings in supramaximal repetitive nerve stimulation with a 10% or more decrease in compound muscle action potential with low-frequency stimuli. 

### 2.2. Methods

Data on patient age at onset, sex, and course of drug treatment were analyzed. The Myasthenia Gravis Foundation of America (MGFA) clinical classification and MGFA post-intervention status (MGFA-PIS) were evaluated at the onset and last visit, respectively. The MGFS-PIS classifies patients as follows: complete stable remission (CSR), pharmacological remission (PR), and minimal manifestations (MM) [14].

Treatment was carried out at the discretion of each attending physician before 2010. Since 2010, the following treatment regimen has been provided within our division. For OMG, oral pyridostigmine was initially administered at 1.5 mg/kg and gradually increased to 4 mg/kg. If the symptoms did not improve adequately for a few weeks, oral prednisolone at a daily dosage of 1.0 mg/kg or high-dose intravenous prednisolone of 30 mg/kg was administered. If the patients developed generalized symptoms, intravenous immunoglobulin was considered before administering prednisolone, as some patients with GMG showed deterioration in the early phase after administration of corticosteroids [15]. In cases where GMG was initially diagnosed, pyridostigmine should be avoided, and treatment should commence with prednisolone. After successfully controlling symptoms for at least 3 months, a one-year tapering of prednisolone is recommended. If the prednisolone response is inadequate or if the patient’s condition worsens while tapering prednisolone, immunosuppressants should be administered, including tacrolimus or mizoribine. 

We also analyzed the drug administration period which was defined as the period from the day of drug treatment to the day it ended. Specifically, three distinct administration periods were identified: those involving the administration of all types of drugs, immunosuppressants, and prednisolone at dosages exceeding 0.35 mg/kg per day. The adverse effects of prednisolone were extracted from medical records. Short stature was defined as height less than 2 standard deviations.

We examined the relationship between the acetylcholine receptor antibody (AChR-Ab) peak and treatment duration. AChR-Ab levels were measured by a clinical testing company (SRL Inc., Tokyo, Japan). Statistical analyses were performed using JMP Pro 16 software for Windows (SAS Institute Inc., Cary, NC, USA). Spearman’s rank correlation coefficient was used to compare the relationship between peak AChR-Ab levels and treatment duration. The level of significance was set at *p* < 0.05.

### 2.3. Ethics

This study was approved by the Review Board of Hyogo Medical University (approval number: 3762). Patient written consent was waived due to the analysis of routine, retrospective clinical data.

## 3. Results

The demographic data of this study are presented in Table 1. In total, 19 participants (8 boys, 11 girls) aged 8 months to 14 years (median, 2 years, and 6 months) at onset were identified. Among the 14 cases whose onset was under 5 years of age, 9 cases (64%) were girls. Among the 11 cases with an onset under 3 years of age, 7 cases (64%) were girls. In total, 14 patients (73.7%) were diagnosed with OMG and 5 (26.3%) with GMG. 

The clinical courses of the patients are presented in Table 2. Regarding the initial treatment, 18 of the 19 patients received pyridostigmine. The remaining patient improved without drug treatment (Case 12). Of the 18 patients treated with pyridostigmine, 14 (74%) were subsequently treated with prednisolone because of the insufficient efficacy of pyridostigmine. Among them, eight (42.1%) patients did not respond sufficiently to prednisolone, and immunosuppressants were added, including tacrolimus, mizoribine, and cyclosporine. Five patients, all diagnosed with GMG received gamma intravenous immunoglobulin therapy. 

The initial manifestations in all patients were ocular symptoms, including ptosis, strabismus, and diplopia. Of the 16 patients with ptosis as the initial manifestation, 14 (87.5%) exhibited unilateral symptoms. All of these patients were initially diagnosed with OMG. However, five patients were subsequently diagnosed to have progressed to GMG due to the occurrence of systemic muscle weakness and positive findings in supramaximal repetitive nerve stimulation. AChR-Ab was present in 12 patients, and 2 patients became positive during follow-up. Finally, AChR-Ab was detected in 14 patients (73.7%), with peak levels ranging from 0.3 to 35.4 nmol/L (median, 1.7 nmol/L). Five patients were negative for AChR-Ab, and none were positive for muscle-specific kinase antibodies. 

Eleven patients completed drug treatment, seven patients did not, and one patient did not receive any drug. Regarding the prognosis of the 11 patients who completed treatment, 10 were classified as CSR and one as PR. The patient classified as PR completed the drug treatment but did not reach one year since completion; thus, he was classified as PR. Regarding the prognosis of the seven patients who did not complete treatment, five were classified as PR and two as MM. Therefore, the patients included in this analysis exhibited either no symptom or only minimal symptoms of functional limitations from MG. The duration from the initiation of drug treatment to reaching the status of MM or PR ranged from 6 to 458 days (median, 55 days).

Among the patients who completed drug treatment, the duration of drug treatment ranged from 11 to 100 months (median, 47 months). In the six patients treated with continuous administration of prednisolone or immunosuppressants, including tacrolimus, mizoribine, and cyclosporine, the treatment duration ranged from 33 to 99 months (median, 56 months). Of the seven patients who did not complete drug treatment, the duration ranged from 34 to 331 months (median, 56 months), and that of prednisolone or immunosuppressants ranged from 33 to 153 months (median, 50 months). 

In the 13 patients where the prednisolone dose per body weight was determined, the median duration of prednisolone treatment with dosing > 0.35 mg/kg/day was 7 months (2–10 months). The adverse effects of prednisolone included hirsutism, mood swings, moon faces, and short stature, and none of the patients experienced severe adverse effects requiring hospitalization. Analysis of the relationship between the peak level of AChR-Ab and treatment duration was performed on eight patients who were positive for AChR-Ab and who did complete treatment, which exhibited no statistical significance (*p* = 0.25).

## 4. Discussion

This study demonstrates the treatment duration required for juvenile MG. All patients treated with pyridostigmine alone had OMG. Of the patients who have completed the drug treatment, treatment with prednisolone or immunosuppressants required at least 33 months of treatment. These results will help develop protocols for treating juvenile MG.

A nationwide study in Japan reported an estimated 20,000 or more patients with MG, and the prevalence at all ages was 11.8 per 100,000, with a peak incidence of 7.0% in children under 5 years of age [16]. Regarding sex differences, it has been reported that 70% of patients are girls, and this is particularly noticeable in children under 3 years of age [16]. In this study, consistent with previous reports, 74% of the cases showed onset under 5 years of age, of which 58% were girls, and under 3 years of age at onset, of which 64% were girls. Racial differences were noted in MG prevalence, with Asian populations exhibiting higher rates than Caucasian populations [17]. Despite these differences, MG prevalence remains similarly low in children across racial groups, posing challenges for developing treatment guidelines.

Juvenile MG predominantly manifests as the ocular type, particularly in younger patients, with approximately 80% of cases occurring in those under 5 years old, compared to around 30% in adults [16,18]. However, OMG may progress to GMG [19]. In their large cohort study, Yang et al. reported that 19 of 161 cases of juvenile OMG progressed to GMG with a median duration of 68 months [5]. They also noted that over 50% of the patients progressed to GMG in less than 2 years. In another cohort study of 62 patients with OMG, the mean follow-up period was 95 months (range, 6–226 months), and 12 patients (19.4%) progressed to GMG [6]. In our study, all patients showed only ocular symptoms and were diagnosed with OMG; eventually, five cases (26.3%) progressed to GMG. The analysis of the factors which related to progression to GMG, including gender, age of onset, AChR-Abs status, revealed no correlation between them [5]. Therefore, the cause of its progression is still not well understood. Although there is inadequate evidence for a formal recommendation, corticosteroids or immunosuppressants should be considered in patients who start showing GMG symptoms [20].

Pyridostigmine is the recommended first-line drug in OMG [12,20,21]. When pyridostigmine fails to provide adequate relief, corticosteroids or immunosuppressants are second-line treatments [12,21]. Corticosteroids are the most widely used drugs for MG; however, they must be used with caution in children because of their adverse effects, including growth failure, poor bone mineralization, and delays in live vaccinations [12]. Therefore, steroid-sparing immunosuppressants should be considered when corticosteroids are ineffective or not tolerated [12,13]. One of the most critical issues is the lack of established evidence regarding the duration of administration of corticosteroids and immunosuppressants. Information on the duration of maintenance, dose reduction strategies, and withdrawal protocols for drug treatment was unavailable in our dataset. According to the regimen in our hospital since 2010, if drug treatment is effective and there is no recurrence, treatment is expected to be completed in approximately 1.5 years to 2 years. However, as has been reported [7,8,9], for some patients, the effects of the drugs are insufficient, or the patient has repeated recurrences, necessitating a more extended period of drug administration. Therefore, the duration from initial treatment to a patient reaching either no symptom or only minimal symptoms of functional limitations from MG varies, and this variability was also observed in our data. Regarding the period until treatment is completed, our study required a minimum treatment period of 33 months, with a median treatment period of 56 months. Therefore, we suggest immunosuppressive therapy should be continued for approximately 3 years. In this study, seven cases did not complete drug treatment yet. However, immunosuppressive therapy for all these cases took more than 33 months. This is consistent with data in patients with completed drug treatment.

The adverse effects of corticosteroids are a major concern, particularly in children. Systemic administration of corticosteroids is used for various diseases, including MG; however, reports on the optimal dose and duration that minimize adverse effects are limited. Past reports have shown that the daily dose of corticosteroids and duration of administration are associated with adverse effects [22,23]. In pediatric patients, growth impairment due to corticosteroids is a particular concern. Rivkees et al. reported that linear growth did not increase with a prednisone dose greater than 0.35 mg/kg daily [24]. To avoid these adverse effects, it is crucial to consider reducing the daily dose or alternating day administration [25]. In a cohort study of 135 patients [26], prednisolone was administered to 79 patients; 1 patient each developed Cushing’s syndrome, weight gain, and hypertension, and 2 patients showed worsening muscle weakness. Their regimen of prednisolone administration was after the performance of high-dose prednisolone pulse therapy; the prednisolone dose was thus tapered to 1.5–2 mg/kg/day. After maintaining it for 4–6 weeks, it was reduced to 0.5 mg/kg every 2 weeks. Although the duration of prednisolone administration was not mentioned in any case, no serious adverse effects were observed. The treatment regimen aligns with the regimen used by our division since 2010, and the patients in our study experienced no severe adverse effects or short stature. We believe that this regimen is appropriate for preventing adverse effects.

The concentration of AChR-Ab cannot predict the severity of clinical symptoms of MG but correlates with individual clinical symptoms [27,28]. In this study, we found no relationship between the duration of drug treatment and AChR-Ab concentration. This result suggests that AChR-Ab concentration does not predict treatment duration or severity.

This study had some limitations. First, as this was a retrospective study with a small number of cases in a single hospital, and the treatment methods varied depending on the attending physician, the results require careful interpretation. Consequently, performing high-quality statistical analysis regarding the treatment period proved challenging. To obtain data with a high level of evidence, we should conduct a randomized control study with a larger sample size; however, the rarity of the disease and its lengthy treatment duration pose challenges. Second, not all patients completed the treatment. Therefore, the shortest treatment duration was 33 months; however, the median and maximum durations may be longer. Juvenile MG is a rare disease that requires a prolonged treatment period. Further investigations, including collaboration with other facilities, are needed to establish appropriate treatment regimens and biomarkers that can predict severity and prognosis.

## Figures and Tables

**Table 1:** Baseline and demographic characteristics.

Patients enrolled, n	19
Age at onset, median (range)	2 y 6 m (0 y 8 m–14 y 5 m)
Duration of follow-up, months: median (range)	50 (2–332)
Sex, n (%)	
Male	8 (42.1)
Female	11 (57.9)
Clinical form, n (%)	
Ocular MG	14 (73.7)
Generalized MG	5 (26.3)
Initial manifestation, n (%)	
Ptosis	16 (84.2)
Unilateral	14
Bilateral	2
Strabismus or diplopia	3 (15.8)
Status in AChR-Ab, n (%)	
Ocular MG	
Positive	11 (78.6)
Negative	3 (21.4)
Generalized MG	
Positive	3 (60)
Negative	2 (40)
Peak level of AchR-Ab in positive patients (nmol/l), median (range)	1.7 (0.3–35.4)

**Table 2:** Characteristics of the patients and duration of the drug treatment.

Case	Sex	Age of Onset	Prognosis	Peak Level of AchR-Ab (nmol/L)	Form	Treatment	Status of Drug Treatment	Duration of Drug Treatment, Months	Duration of Treatment with Prednisolone and Immunosuppressants, Months	Duration of Prednisolone Treatment with Dosing > 0.35 mg/kg/day, Months
1	M	2 y 2 m	PR	1.9	OMG	Py, PSL	completed	100	99	7
2	M	2 y 4 m	CSR	1	OMG	Py, PSL, MZR	completed	33	33	no data
3	M	2 y 6 m	CSR	2.1	OMG	Py, PSL, Tac	completed	49	48	4
4	F	2 y 6 m	CSR	N.D.	OMG	Py, PSL, Tac	completed	56	56	4
5	F	3 y 2 m	CSR	0.4	OMG	Py, PSL, Tac	completed	36	35	2
6	F	3 y 5 m	CSR	0.78	OMG	Py	completed	11	-	-
7	M	4 y 1 m	CSR	3.8	OMG	Py	completed	44	-	-
8	M	8 y 6 m	CSR	N.D.	OMG	Py, PSL, Tac	completed	58	57	8
9	M	10 y 2 m	CSR	0.3	OMG	Py	completed	18	-	-
10	M	14 y 5 m	CSR	0.7	OMG	Py	completed	47	-	-
11	F	0 y 11 m	CSR	N.D.	GMG	Py, PSL, IVIG, Tac	completed	64	62	8
12	F	7 y 5 m	CSR	N.D.	OMG	no drug treatment	none	-	-	-
13	F	2 y 2 m	PR	1.7	OMG	Py, PSL	ongoing	40	39	10
14	F	2 y 10 m	PR	0.4	OMG	Py, PSL	ongoing	56	54	6
15	F	8 y 9 m	MM	0.4	OMG	Py, PSL	ongoing	60	50	4
16	M	0 y 8 m	PR	N.D.	GMG	Py, PSL, IVIG, Tac	ongoing	59	56	7
17	F	0 y 11 m	MM	6.5	GMG	Py, PSL, IVIG	ongoing	44	43	9
18	F	1 y 8 m	PR	11	GMG	Py, PSL, IVIG	ongoing	34	33	7
19	F	2 y 0 m	PR	35.4	GMG	Py, PSL, CyA, IVIG, thymectomy	ongoing	331	153	4

Note: OMG: ocular-type myasthenia gravis, GMG: generalized-type myasthenia gravis, CSR: complete stable remission, PR: pharmacological remission, MM: minimal manifestations, Py: pyridostigmine, PSL: prednisolone, Tac: tacrolimus, MZR: mizoribine, CyA: cyclosporine, IVIG: intravenous immunoglobulin therapy, N.D.: not detected.

## Data Availability

The original contributions presented in the study are included in the article, and further inquiries can be directed to the corresponding author.
